# Breathing Biofeedback for Police Officers in a Stressful Virtual Environment: Challenges and Opportunities

**DOI:** 10.3389/fpsyg.2021.586553

**Published:** 2021-03-12

**Authors:** Jan C. Brammer, Jacobien M. van Peer, Abele Michela, Marieke M. J. W. van Rooij, Robert Oostenveld, Floris Klumpers, Wendy Dorrestijn, Isabela Granic, Karin Roelofs

**Affiliations:** ^1^Behavioural Science Institute, Radboud University, Nijmegen, Netherlands; ^2^Donders Institute for Brain, Cognition and Behaviour, Radboud University, Nijmegen, Netherlands; ^3^NatMEG, Department of Clinical Neuroscience, Karolinska Institutet, Stockholm, Sweden; ^4^Police Academy of the Netherlands, Apeldoorn, Netherlands; ^5^Faculty of Law, Radboud University, Nijmegen, Netherlands

**Keywords:** biofeedback, virtual reality, stress exposure, user experience, physiological computing

## Abstract

As part of the Dutch national science program “Professional Games for Professional Skills” we developed a stress-exposure biofeedback training in virtual reality (VR) for the Dutch police. We aim to reduce the acute negative impact of stress on performance, as well as long-term consequences for mental health by facilitating physiological stress regulation during a demanding decision task. Conventional biofeedback applications mainly train physiological regulation at rest. This might limit the transfer of the regulation skills to stressful situations. In contrast, we provide the user with the opportunity to practice breathing regulation while they carry out a complex task in VR. This setting poses challenges from a technical – (real-time processing of noisy biosignals) as well as from a user-experience perspective (multi-tasking). We illustrate how we approach these challenges in our training and hope to contribute a useful reference for researchers and developers in academia or industry who are interested in using biosignals to control elements in a dynamic virtual environment.

Acute physiological stress impairs performance by causing deficits in motor control, cognition, or perception (Nieuwenhuys et al., 2009; Nieuwenhuys and Oudejans, 2010; Andersen and Gustafsberg, 2016) and can negatively impact mental health in the long term (Maguen et al., 2009). By teaching acute stress regulation, biofeedback could help preserve performance in challenging situations, and lessen the detrimental impact of repeated stress responses (Andersen and Gustafsberg, 2016). Since police are frequently confronted with demanding situations that require quick, high-stakes decisions, police forces, including the Dutch police, recently started introducing biofeedback to their training curricula (van der Meulen et al., 2018). However, the physiological regulation skills are usually exclusively taught at rest which might limit their transfer to stressful situations (Bouchard et al., 2012).

To make physiological regulation skills more robust to degradation under stress, we developed a training that combines biofeedback with a demanding task in virtual reality (VR). VR is increasingly used for stress-exposure training since it offers the opportunity to create immersive and stressful, yet controlled environments (Pallavicini et al., 2016). However, to date, the majority of biofeedback trainings do not leverage the potential of VR (Jerčić and Sundstedt, 2019). While providing a more immersive environment than screen-based biofeedback applications, current VR biofeedback applications require the user to stay relatively motionless and to solely focus on the biofeedback (van Rooij et al., 2016; Rockstroh et al., 2019). In contrast, we provide police with the opportunity to recognize and regulate their physiological stress response *while* they carry out a demanding task in a stressful environment. Specifically, we provide an environment that requires the user to regulate their breathing while making fast decisions based on ambiguous, constantly changing information. We will refer to this kind of biofeedback as stress-exposure biofeedback. The promise of stress-exposure biofeedback has already been demonstrated in a military population, albeit in a non-VR setting (Bouchard et al., 2012).

Compared to conventional biofeedback applications, stress-exposure biofeedback introduces challenges from a technical and user-experience perspective. Here, we summarize and illustrate challenges in three critical areas: (1) the choice of a biofeedback parameter, (2) the implementation of the biofeedback processing, and (3) the biofeedback representation in the virtual environment. We hope to demonstrate the feasibility of stress-exposure biofeedback by illustrating each of these challenges with our implementation. Further, by sharing our experiences, decisions, and considerations we hope to contribute a useful reference for researchers and developers in academia or industry who are interested in using physiological signals to control elements in a dynamic virtual environment.

## What Is Biofeedback?

People are usually not conscious of their autonomic physiology, let alone able to regulate it (Price and Hooven, 2018). Biofeedback reveals internal physiological processes and provides guidance on how to change them, which can reduce anxiety and facilitate coping with stress (Yu et al., 2018; Tolin et al., 2020). In the following, we discuss how a conventional biofeedback application works and then show how it can be adapted for stress-exposure biofeedback.

**FIGURE 1:**
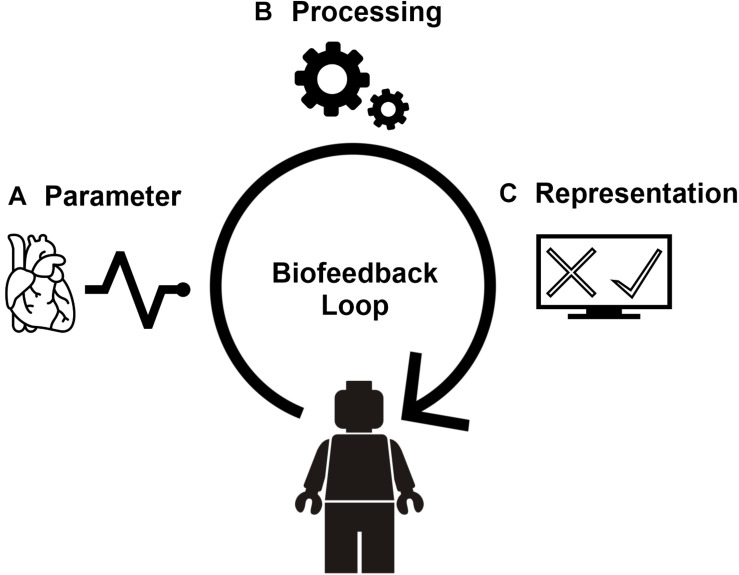
The biofeedback loop. Biofeedback is a form of human-computer interaction which puts the user in a closed real-time loop. We divide this loop in three components that are discussed throughout the paper. First, a biofeedback parameter **(A)** is extracted from a physiological modality. For example, heart rate derived from an electrocardiogram. Second, in a series of processing steps **(B)** the current state of the parameter is evaluated relative to a target state. The processing results in a biofeedback score that expresses how well the parameter’s current state matches the target state. The biofeedback score can be qualitative (match vs. no match) or quantitative (degree of matching). Finally, the biofeedback score is translated into a representation **(C)** consisting of (a combination of) visual, auditory, or tactile feedback which can be embedded in a variety of media, such as VR.

Let’s consider an example of a trainee who is taught to downregulate their heart rate (i.e., the biofeedback parameter, Figure 1A). Electrocardiogram electrodes measure the electrical activity of the heart, which is send to a processing unit (Figure 1B). The unit estimates the trainee’s heart rate and applies a decision criterion that determines if the heart rate increased or decreased compared to the last measurement. The decision criterion is based on a biofeedback target, for example a decrease of 10%. Finally, the biofeedback representation reveals the outcome of the biofeedback processing (Figure 1C): for example, a green screen in case of a decrease, a red screen in case of an increase, or a blue screen if no change occurred. By rewarding the downregulation of the heart rate, the biofeedback system guides the trainee to the target through operant learning (Weerdmeester et al., 2020).

In the calm environments of conventional biofeedback applications, the trainee can fully focus on regulating their physiology to meet the biofeedback target. However, in a more stressful, demanding context, this is no longer possible since the environment might distract the user from the physiological regulation. Additionally, stress-exposure biofeedback poses technical challenges since acquiring and processing physiological signals is more challenging in dynamic conditions compared to resting conditions. That is, stress-exposure biofeedback creates additional demands both for the user and the developer. We will discuss these demands based on our application, in the context of the three challenges mentioned before: (1) the choice of the biofeedback parameter, (2) the real-time processing of the biofeedback parameter (evaluating match with biofeedback target), and (3) the representation of the biofeedback in the training environment.

## Challenge 1: Choice of the Biofeedback Parameter

### Prioritizing Controllability

To account for the trainee’s divided attention during stress-exposure biofeedback, their control of the biofeedback parameter should be as easy and direct as possible. A variety of physiological modalities are related to stress and can serve as a basis for a biofeedback parameter, such as electroencephalography, heart rate variability or breathing (Yu et al., 2018; Tolin et al., 2020). These modalities differ in terms of their controllability and one of the easiest-to-control physiological modalities is breathing (Nacke et al., 2011; Parnandi and Gutierrez-Osuna, 2019). This is why we chose breathing rate as our biofeedback parameter, with a biofeedback target of 4 to 12 breaths per minute (Russo et al., 2017), which is considerably lower than human breathing rates under cognitive or physical load (Nicolò et al., 2017; Hidalgo-Muñoz et al., 2019). Slow breathing affects the autonomic nervous system by increasing vagus nerve activity and evoking a shift toward parasympathetic dominance (Russo et al., 2017). This might help regulate physiological arousal in an emotionally or cognitively challenging situation. In summary, breathing seems to offer both controllability and the ability to regulate physiological arousal. We evaluated the controllability of the biofeedback parameter and the achievability of the biofeedback target in a sample of nine police trainers. Each of them completed 10 training sessions over the course of three weeks. Each session lasted about 15 min and was played with or without biofeedback. Sessions with and without biofeedback were alternated in order to get an impression of the transfer of the physiological regulation skill.

**FIGURE 2:**
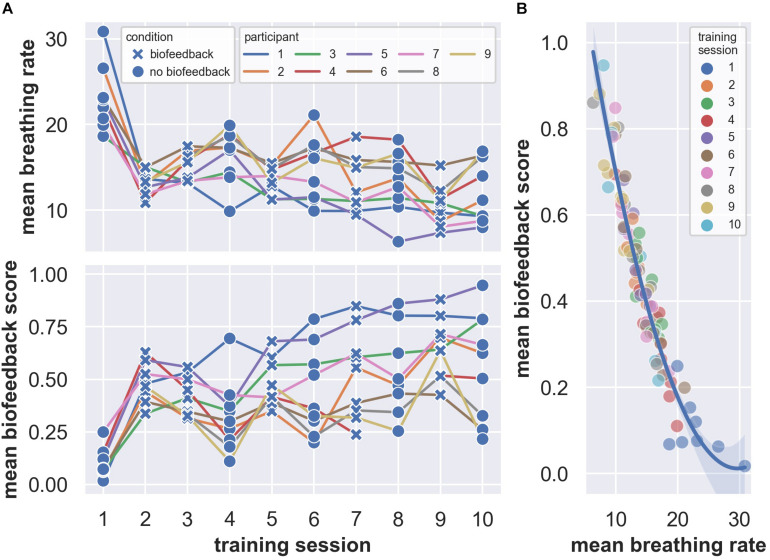
Mean breathing rates **(A, upper panel)** and biofeedback scores **(A, lower panel)** over training sessions (alternating with and without biofeedback). **(B)** Quadratic fit characterizing the relationship of the session means of breathing rate and biofeedback score. The shaded region indicates a bootstrapped 95% confidence interval for the quadratic fit.

**FIGURE 3:**
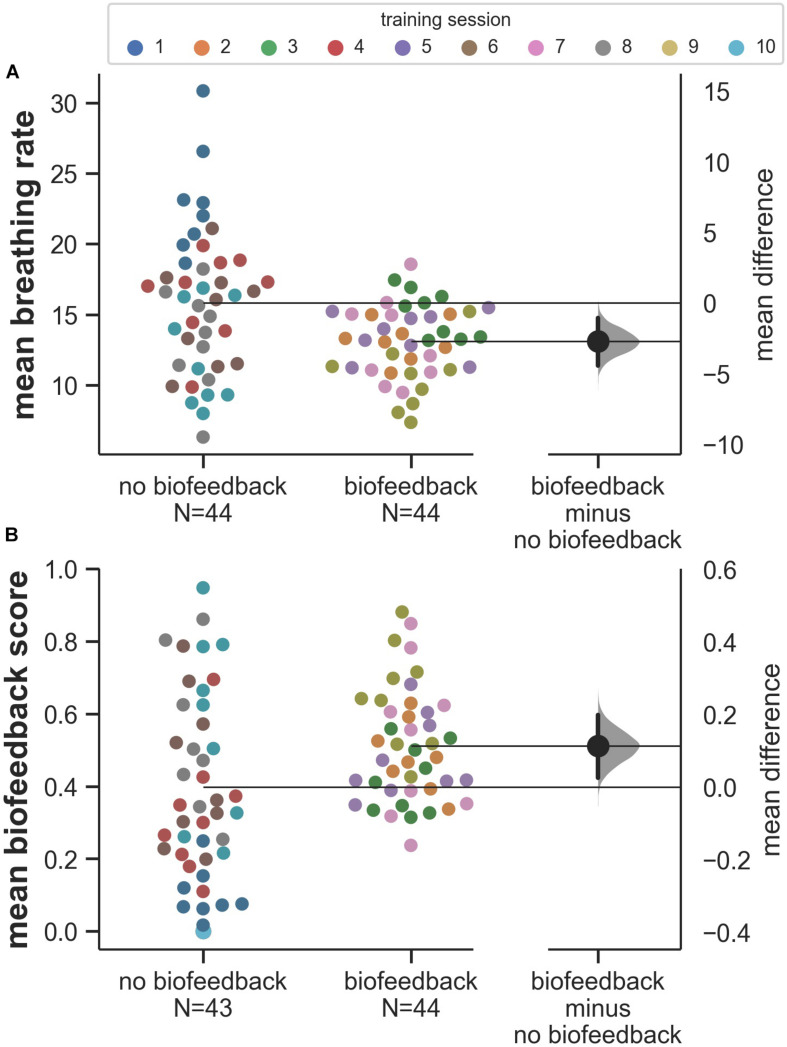
Comparison of mean breathing rates **(A)** and biofeedback scores **(B)** in sessions with and without biofeedback. In sessions without biofeedback, the biofeedback score was computed and recorded but did not affect the game. The distributions on the right side of panels **(A,B)** display bootstrapped 95% confidence intervals for the mean differences between the conditions (Ho et al., 2019). Note that the condition differences are mainly driven by the first training session.

The pilot data suggest that the biofeedback parameter is controllable and that the biofeedback target is achievable in a stress-exposure context. We observed that mean breathing rates decrease over sessions (Figure 2A, upper panel) and are lower in biofeedback sessions compared to sessions without biofeedback (Figure 3A). Many of the mean breathing rates fall within the biofeedback target range of 4 to 12 breaths per minute (e.g., Figure 3A). Similarly, participants continuously improve their biofeedback scores over the training sessions (Figure 2A, lower panel) and their mean biofeedback scores are higher in biofeedback sessions compared to sessions without biofeedback (Figure 3B, see challenge 2 for details on the biofeedback score). Moreover, the decreasing trend in breathing rate and increasing trend in the biofeedback score shown in Figure 2A do not seem to merely reflect the participants’ habituation to the stressful environment. This is evident by the biofeedback-induced session-by-session fluctuations on top of the decreasing- (Figure 2A, upper panel) or increasing trend (Figure 2A, lower panel). These fluctuations seem to be an indication that, following biofeedback sessions, participants transfer the physiological regulation skill to subsequent sessions without biofeedback. Finally, we found the session averages of breathing rates and biofeedback scores to be strongly related (Figure 2B). This indicates that the biofeedback score is a valid representation of the extent to which participants manage to achieve the biofeedback target.

### The Costs of Controllability

However, there are downsides to choosing a biofeedback parameter that is easy to control. As illustrated earlier (Figure 1), biofeedback is a form of human-computer-interaction (HCI). The human in the HCI is used to *immediate* and *invariable* control over the computer (Limerick et al., 2014; Attig et al., 2017). If someone presses the “k” key on their keyboard they expect the letter to appear on the screen instantaneously (immediacy). Also, they expect the letter to always be “k,” not “o” occasionally (invariability). Immediacy or invariability cannot be guaranteed in a biofeedback system.

#### Variability Is More Noticeable

The non-deterministic nature of human physiology introduces variability to the biofeedback system. Consider the example of heart rate downregulation again: At some point the trainee may notice that they can decrease their heart rate by exhaling deeply. However, two identical outbreaths (in terms of duration and depth) don’t necessarily produce the same decrease in heart rate. In general, a biofeedback parameter cannot be controlled in a deterministic manner. That is, even if a trainee consistently applies a specific regulation strategy, they will achieve variable outcomes in terms of the behavior of the biofeedback parameter. Variability is a greater challenge for biofeedback parameters that are under more direct control, because the trainee will have a clearer sense of their current physiological state. Consequently, they will more easily notice variability-induced discrepancies between their perceived physiological state and the biofeedback representation.

#### Delay Is More Noticeable

The biofeedback processing introduces a noticeable delay between the recording of the biosignal and the presentation of the feedback. To get a reliable and accurate estimate of a physiological state, it usually has to be integrated over longer time windows. For example, estimating instantaneous breathing rate requires at least a full breathing cycle and several breathing cycles have to be averaged to obtain a reliable and accurate estimate in the presence of measurement artifacts. Consequently, the biofeedback presented to the trainee will not pertain to their instantaneous physiological state. This can violate the trainee’s expectation of immediacy, especially during abrupt shifts in the physiological state. For example, when someone shifts from slow, deep breathing to a markedly faster breathing rhythm or vice versa, the response of the biofeedback representation can appear sluggish.

In summary, stress-exposure biofeedback benefits from a biofeedback parameter that is relatively easy to control. However, controllability comes at the cost of more salient variability and delay. Nevertheless, we think that controllability outweighs these costs especially since both variability and delay can partly be alleviated during biofeedback processing (Challenge 2) and the careful design of the biofeedback representation (Challenge 3).

## Challenge 2: Implementation of the Biofeedback Processing

The goal of biofeedback processing is to map the biofeedback parameter to the biofeedback representation. This involves two steps: First, the current state of the biofeedback parameter (e.g., breathing rate) has to be estimated. Second, the extent to which the current state of the biofeedback parameter approaches the biofeedback target has to be evaluated (e.g., breathing rate between 4 and 12 breaths per minute). Based on how closely the trainee matches the target, we then compute a biofeedback score that is ultimately reflected in the biofeedback representation (see Challenge 3). Supplementary Material 1 contains details on the hardware and software used for the biofeedback processing.

### Estimating the Current State of the Biofeedback Parameter

The first processing step is to estimate the current breathing rate from the raw data, which comes from a breathing belt around the trainee’s lower abdomen. The raw data contains a phasic pattern with inhalation peaks and exhalation troughs, and conceptually, the instantaneous breathing rate is based on the temporal difference between moments of the same phase (e.g., inhalation peaks or exhalation troughs). Supplementary Material 2 contains a detailed description of how we estimate breathing rate.

Unfortunately, the raw sensor data does not exclusively reflect the dynamics of the biofeedback parameter. Instead, it contains artifacts that can originate from the measurement environment or unrelated physiological activity. For example, our breathing belt tracks breathing by measuring changes in torso circumference. However, since the trainee is standing and moving their upper body, the data contains movement artifacts that are in the same frequency range as (fast) breathing and cannot easily be filtered out (Supplementary Material 2 and Supplementary Figure 4). Regardless of the physiological modality, artifacts tend to be more prevalent in stress-exposure biofeedback compared with biofeedback at rest and from a user-experience perspective they contribute to both the problem of variability and delay (see Challenge 1).

Artifacts increase variability, which can frustrate the trainee because it can make the biofeedback target seem unattainable. This problem can be alleviated by making the biofeedback target less specific. When the target range is narrow (e.g., breathing at 6 breaths per minute), the estimated breathing rate will more often be “off target” due to artifacts. In contrast, when the target range is broader, the influence of artifacts is less perceptible since the wider margin compensates for artifact-induced variability in the estimated breathing rate. However, if the target range is too broad the training goal can lose specificity from a user’s perspective.

Additionally, artifacts can increase delay. The presence of artifacts makes estimating the breathing rate from a short segment of data unreliable due to a low signal-to-noise-ratio. To increase the signal-to-noise-ratio, longer segments need to be processed (Hassan and Anwar, 2010). However, this means that at each point in time, the current estimate of the breathing rate and corresponding biofeedback representation do not exclusively reflect the most recent physiological state. Therefore, there is a trade-off between delay and the reliability of the biofeedback: More reliable estimates of breathing rate from longer segments come at the cost of more delay. A good compromise between reliability and delay allows for a reliable estimation of the biofeedback parameter from a technical perspective, while still feeling relatively responsive to changes in the biofeedback parameter from a user’s perspective.

### Comparing the Current State of the Biofeedback Parameter to the Biofeedback Target

In a second step, the biofeedback processing quantifies how much the current state of the biofeedback parameter matches the biofeedback target. This matching is then expressed quantitatively (e.g., percentage) or qualitatively (e.g., binary) in the form of a biofeedback score. The computation of the biofeedback score can differ widely between biofeedback applications. However, regardless of the specific application, the computation of the biofeedback score presents the developer with seemingly small choices regarding algorithmic parameters that can profoundly influence the user experience. For example, related to our application, we already mentioned choosing the upper and lower bound of the biofeedback target range. We illustrate additional parameter choices related to the computation of the biofeedback score in Supplementary Material 2. Making these choices based on iterative user testing is crucial to ensure a satisfactory user experience (Scholten and Granic, 2019). User testing is greatly facilitated by the ability to visualize the raw data and intermediate processing steps as well as the ability to adjust parameters in real-time. Therefore, we implemented a dashboard that allowed us to fine-tune the biofeedback processing in real-time to immediately experience the effects of different parameter settings (Supplementary Material 2 and Supplementary Figure 3).

## Challenge 3: Biofeedback Representation in the Virtual Environment

Finally, the biofeedback score needs to be presented to the trainee in a meaningful and intuitive way. In our virtual environment, the trainee finds themselves at the center of a poorly lit parking garage where they are surrounded by zombies that can either be benign or hostile, which is indicated by their eye-color or body shape (Supplementary Material 3 and Supplementary Figure 5). These indicators change several times throughout the training, which is announced via radio dispatch calls that mimic a suspect description. The trainee has to shoot the hostile zombies while leaving the benign zombies unharmed. In collaboration with our advisors at the Dutch police, we made an effort to steer clear of the “shoot ‘em up” genre of video games (i.e., reflexive shooting at uniformly hostile adversaries) by designed the shooting task such that the player is primed to make, careful, deliberate shooting decisions. Additionally, the task engages behaviors that are universally relevant to police: The trainee has to rely on good situational awareness, be constantly vigilant to changes in information, and be able to override response biases by flexibly incorporating these changes in their decisions (Di Nota and Huhta, 2019). At the same time, by eliciting police-relevant behavior in an overtly fictional environment, we sidestep the necessity to simulate realistic police incidents and avoid overtraining the officers to idiosyncratic elements of a realistic simulation (Michela et al., 2019).

**FIGURE 4:**
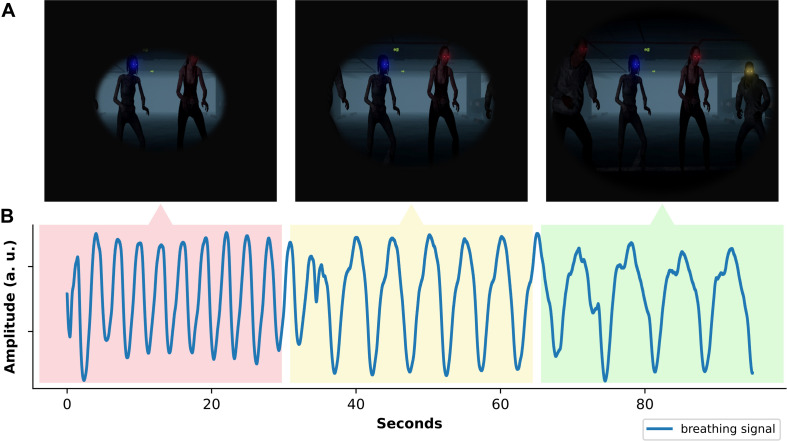
Biofeedback representation and its relation to breathing. The peripheral vision in the VR headset **(A)** responds to the user’s breathing rate. **(B)** Shows the raw breathing signal of a user who transitions from a fast breathing rate to a slower breathing rate that matches the biofeedback target more closely.

In this environment the biofeedback representation needs to be as salient and intuitive as possible, since regulating physiology becomes part of a multi-tasking exercise. The player has to allocate cognitive resources to both the decision task as well as the physiological regulation, which can worsen performance on both tasks (Wickens, 2002). Additionally, high task demands in a multitasking context can increase heart- and breathing rates (Fairclough et al., 2005). Together, these findings suggest that deliberate physiological regulation may be especially challenging in a multitasking context. Further, the multi-tasking bears the danger that the biofeedback is misattributed to behavior instead of the physiological regulation. For example, the trainee might attribute a poor biofeedback score to shooting a benign zombie rather than their fast breathing. This misattribution can be prevented by presenting the biofeedback such that it intuitively represents physiology in the task context. We use the analogy of tunnel vision which is relatable for Dutch police since they are introduced to this concept during their academy training (van der Meulen et al., 2018). Figure 4 illustrates how the trainee’s peripheral vision widens as the breathing gets slower and deeper.

This is particularly salient since the decision task requires the trainee to monitor all 360° of their surroundings, which makes losing peripheral vision costly. The tunnel vision is amplified by modulating the brightness of environmental lights. In general, the biofeedback representation should intuitively fit into the context and training goals of the application to facilitate immersion and engagement. This can lead to fundamentally different representations of the same biofeedback parameter. For example, DEEP, another breathing biofeedback training in VR, teaches the user to leverage their exhalation to propel themselves forward and their inhalation to float upward in a virtual underwater environment (van Rooij et al., 2016).

Moreover, to ease multi-tasking, the biofeedback representation should only interfere with game play if this is intended (such as the tunnel vision), not for ergonomic reasons such as graphs that are placed inconveniently in the trainee’s field of vision. In the same vein, we chose to not include a more explicit biofeedback representation such as the commonly used statistical graphs (Sun et al., 2017) to avoid burdening the trainee with monitoring yet another element in the environment.

Another crucial element of the biofeedback representation is its stepwise introduction. Before the trainee enters our multi-tasking environment, they are guided through a breathing tutorial that gradually introduces them to the regulation skill. The trainee starts with a breathing exercise that requires them to breathe along with a visual pacer (Supplementary Material 3 and Supplementary Figure 6A). Once they feel comfortable with the breathing skill, we demonstrate the effects of the breathing regulation. This demonstration includes an explicit component (bar graph) providing clear feedback on the current physiological state as well as the more implicit environmental effects described earlier (tunnel vision and environmental lights) (Supplementary Material 3 and Supplementary Figure 6B). Note that the latter are implicit only in terms of the concrete representation, not in terms of saliency. Once the trainee has a good understanding of how their breathing affects the environment, we remove the bar graph and they practice the regulation skill in a simplified version of the decision task (Supplementary Material 3 and Supplementary Figure 6C) before entering the full-fledged training. The gradual introduction of the regulation skill in progressively more challenging contexts avoids overwhelming the trainee with the demands of multi-tasking and is believed to facilitate skill transfer (Driskell and Johnston, 1998; Driskell et al., 2001).

Lastly, in designing the biofeedback representation, it is helpful to know the behavior of the biofeedback parameter as early as possible. This includes being familiar with its temporal dynamics as well as extreme patterns. Regarding the latter, it is useful to account for the possibility that trainees “get stuck” in a physiological state and consequently struggle to meet the biofeedback target. In this scenario, it is important for the biofeedback representation to be designed such that the trainee can still function in the environment. For example, in our environment the trainee always retains a minimum of visibility even with the worst biofeedback score (see Figure 4).

## Discussion

Designing and implementing a stress-exposure biofeedback training requires a re-thinking of conventional biofeedback training. This introduces challenges around (1) the choice of a biofeedback parameter, (2) the biofeedback processing, and (3) the representation of the biofeedback. We examined these challenges from both a technical as well as a user-experience perspective and illustrated the feasibility of stress-exposure biofeedback with examples from a breathing-based stress regulation training for police.

We highlighted the importance of controllability of the biofeedback parameter as well as the attainability of the biofeedback target. Additionally, we showed how seemingly small algorithmic decisions during the real-time computation of the biofeedback can have far-reaching consequences for the user experience, and emphasized the importance to arrive at these decisions during iterative user testing. Finally, we point out the relevance of a salient, intuitive biofeedback representation that is introduced gradually, and tailored to the task context and goals.

In demonstrating the feasibility of stress-exposure biofeedback, we hope to advance this biofeedback paradigm and to help pave the way for studies that explore its potential to diminish the short- and long-term consequences of repeated stress-exposure. Above all, we hope this paper and its Supplementary Material provide a useful reference for developers and researchers in academia or industry who are interested in using physiological signals to control elements in a dynamic virtual environment.

## Ethics Statement

The studies involving human participants were reviewed and approved by the Ethics Committee Faculty of Social Sciences, Radboud University, Nijmegen, Netherlands. Written informed consent for participation was not required for this study in accordance with the national legislation and the institutional requirements. Written informed consent was obtained from the individual(s) for the publication of any potentially identifiable images or data included in this article.

## Author Contributions

JB wrote the manuscript, conceptualized and implemented the biofeedback, and analyzed the pilot data. RO conceptualized and implemented the IT infrastructure (software and hardware) for the biofeedback. RO and FK supervised the implementation of the biofeedback. JB, JP, MR, FK, AM, IG, KR, and WD conceptualized the VR environment and decision task. WD coordinated the pilot data collection. AM planned and conducted the pilot data collection. All authors reviewed and contributed to the manuscript.

## Conflict of Interest

The authors declare that the research was conducted in the absence of any commercial or financial relationships that could be construed as a potential conflict of interest.

## References

[B1] Andersen J. P. Gustafsberg H. (2016). A training method to improve police use of force decision making: a randomized controlled trial. *SAGE Open* 6:215824401663870. 10.1177/2158244016638708

[B2] Attig C. Rauh N. Franke T. Krems J. F. (2017). “System latency guidelines then and now–is zero latency really considered necessary?,” in *Engineering Psychology and Cognitive Ergonomics: Cognition and Design Lecture Notes in Computer Science*, ed. Harris D. (Cham: Springer International Publishing), 3–14. 10.1007/978-3-319-58475-1_1

[B3] Bouchard S. Bernier F. Boivin É Morin B. Robillard G. (2012). Using biofeedback while immersed in a stressful videogame increases the effectiveness of stress management skills in soldiers. *PLoS One* 7:e36169. 10.1371/journal.pone.0036169 22558370 PMC3338628

[B4] Cokelaer T. Hasch J. (2017). “Spectrum”: spectral analysis in Python. *J. Open Source Softw.* 2:348. 10.21105/joss.00348

[B5] Di Nota P. M. Huhta J.-M. (2019). Complex motor learning and police training: applied, cognitive, and clinical perspectives. *Front. Psychol.* 10:1797. 10.3389/fpsyg.2019.01797 31440184 PMC6692711

[B6] Driskell J. E. Johnston J. H. (1998). “Stress exposure training,” in *Making Decisions Under Stress: Implications for Individual and Team Training*, eds Cannon-Bowers J. A. Salas E. (Washington: American Psychological Association), 191–217. 10.1037/10278-007

[B7] Driskell J. E. Johnston J. H. Salas E. (2001). Does stress training generalize to novel settings? *Hum. Factors* 43 99–110. 10.1518/001872001775992471 11474766

[B8] Fairclough S. H. Venables L. Tattersall A. (2005). The influence of task demand and learning on the psychophysiological response. *Int. J. Psychophysiol.* 56 171–184. 10.1016/j.ijpsycho.2004.11.003 15804451

[B9] Hassan U. Anwar M. S. (2010). Reducing noise by repetition: introduction to signal averaging. *Eur. J. Phys.* 31, 453–465. 10.1088/0143-0807/31/3/003

[B10] Hidalgo-Muñoz A. R. Béquet A. J. Astier-Juvenon M. Pépin G. Fort A. Jallais C., et al. (2019). Respiration and heart rate modulation due to competing cognitive tasks while driving. *Front. Hum. Neurosci.* 12:525. 10.3389/fnhum.2018.00525 30687043 PMC6338053

[B11] Ho J. Tumkaya T. Aryal S. Choi H. Claridge-Chang A. (2019). Moving beyond P values: data analysis with estimation graphics. *Nat. Methods* 16 565–566. 10.1038/s41592-019-0470-3 31217592

[B12] Hunter J. D. (2007). Matplotlib: a 2D graphics environment. *Comput. Sci. Eng.* 9 90–95. 10.1109/MCSE.2007.55

[B13] Jerčić P. Sundstedt V. (2019). Practicing emotion-regulation through biofeedback on the decision-making performance in the context of serious games: a systematic review. *Entertain. Comput.* 29 75–86. 10.1016/j.entcom.2019.01.001

[B14] Limerick H. Coyle D. Moore J. W. (2014). The experience of agency in human-computer interactions: a review. *Front. Hum. Neurosci.* 8:643. 10.3389/fnhum.2014.00643 25191256 PMC4140386

[B15] Maguen S. Metzler T. J. McCaslin S. E. Inslicht S. S. Henn-Haase C. Neylan T. C., et al. (2009). Routine work environment stress and PTSD symptoms in police officers. *J. Nerv. Ment. Dis.* 197 754–760. 10.1097/NMD.0b013e3181b975f8 19829204 PMC3974929

[B16] Michela A. Rooij M. M. J. W. Klumpers F. Peer J. M. Roelofs K. Granic I. (2019). Reducing the noise of reality. *Psychol. Inquiry* 30 203–210. 10.1080/1047840X.2019.1693872

[B17] Nacke L. E. Kalyn M. Lough C. Mandryk R. L. (2011). “Biofeedback game design: using direct and indirect physiological control to enhance game interaction,” in *Proceedings of the 2011 annual conference on Human factors in computing systems - CHI ’11* (Vancouver, BC: ACM Press), 103–112. 10.1145/1978942.1978958

[B18] Nicolò A. Massaroni C. Passfield L. (2017). Respiratory frequency during exercise: the neglected physiological measure. *Front. Physiol.* 8:922. 10.3389/fphys.2017.00922 29321742 PMC5732209

[B19] Nieuwenhuys A. Caljouw S. R. Leijsen M. R. Schmeits B. A. J. Oudejans R. R. D. (2009). Quantifying police officers’ arrest and self-defence skills: does performance decrease under pressure? *Ergonomics* 52 1460–1468. 10.1080/00140130903287981 19941180

[B20] Nieuwenhuys A. Oudejans R. R. D. (2010). Effects of anxiety on handgun shooting behavior of police officers: a pilot study. *Anxiety Stress Coping* 23 225–233. 10.1080/10615800902977494 19462309

[B21] Pallavicini F. Argenton L. Toniazzi N. Aceti L. Mantovani F. (2016). Virtual reality applications for stress management training in the military. *Aerosp. Med. Hum. Perform.* 87 1021–1030. 10.3357/AMHP.4596.2016 28323588

[B22] Parnandi A. Gutierrez-Osuna R. (2019). Visual biofeedback and game adaptation in relaxation skill transfer. *IEEE Trans. Affect. Comput.* 10 276–289. 10.1109/TAFFC.2017.2705088

[B23] Price C. J. Hooven C. (2018). Interoceptive awareness skills for emotion regulation: theory and approach of Mindful Awareness in Body-Oriented Therapy (MABT). *Front. Psychol.* 9:798. 10.3389/fpsyg.2018.00798 29892247 PMC5985305

[B24] PyQtGraph (2020). *PyQtGraph - Scientific Graphics and GUI Library for Python.* Available online at: http://www.pyqtgraph.org/ (accessed July 22, 2020).

[B25] Riverbank Computing (2020). *Riverbank Computing | Introduction.* Available online at: https://riverbankcomputing.com/software/pyqt/ (accessed July 22, 2020).

[B26] Rockstroh C. Blum J. Göritz A. S. (2019). Virtual reality in the application of heart rate variability biofeedback. *Int. J. Hum.Comput. Stud.* 130 209–220. 10.1016/j.ijhcs.2019.06.011

[B27] Russo M. A. Santarelli D. M. O’Rourke D. (2017). The physiological effects of slow breathing in the healthy human. *Breathe* 13 298–309. 10.1183/20734735.009817 29209423 PMC5709795

[B28] Scholten H. Granic I. (2019). Use of the principles of design thinking to address limitations of digital mental health interventions for youth: viewpoint. *J. Med. Internet Res.* 21:e11528. 10.2196/11528 31344671 PMC6682276

[B29] SciCrunch (2020). *SciCrunch | Browse RRID Content.* Available online at: https://scicrunch.org/resources/about/registry/SCR_018732 (accessed July 22, 2020).

[B30] Sun Z. Cao N. Ma X. (2017). “Attention, comprehension, execution: effects of different designs of biofeedback display,” in *Proceedings of the 2017 CHI Conference Extended Abstracts on Human Factors in Computing Systems - CHI EA ’17* (Denver, CO: ACM Press), 2132–2139. 10.1145/3027063.3053082

[B31] Tolin D. F. Davies C. D. Moskow D. M. Hofmann S. G. (2020). “Biofeedback and neurofeedback for anxiety disorders: a quantitative and qualitative systematic review,” in *Anxiety Disorders Advances in Experimental Medicine and Biology*, ed. Kim Y.-K. (Singapore: Springer Singapore), 265–289. 10.1007/978-981-32-9705-0_16 32002934

[B32] van der Meulen E. Bosmans M. W. G. Lens K. M. E. Lahlah E. van der Velden P. G. (2018). Effects of mental strength training for police officers: a three-wave quasi-experimental study. *J. Police Crim. Psych.* 33 385–397. 10.1007/s11896-017-9247-8

[B33] van der Walt S. Colbert S. C. Varoquaux G. (2011). The numpy array: a structure for efficient numerical computation. *Comput. Sci. Eng.* 13 22–30. 10.1109/MCSE.2011.37

[B34] van Rooij M. Lobel A. Harris O. Smit N. Granic I. (2016). “DEEP: a biofeedback virtual reality game for children at-risk for anxiety,” in *Proceedings of the 2016 CHI Conference Extended Abstracts on Human Factors in Computing Systems - CHI EA ’16* (San Jose, CA: ACM Press), 1989–1997. 10.1145/2851581.2892452

[B35] Van Rossum G. Drake F. L. (2009). *Python 3 Reference Manual.* Scotts Valley, CA: CreateSpace.

[B36] Virtanen P. Gommers R. Oliphant T. E. Haberland M. Reddy T. Cournapeau D., et al. (2020). SciPy 1.0: fundamental algorithms for scientific computing in python. *Nat. Methods* 17 261–272. 10.1038/s41592-019-0686-2 32015543 PMC7056644

[B37] Waskom M. Botvinnik O. Gelbart M. Ostblom J. Hobson P. Lukauskas S., et al. (2020). mwaskom/seaborn: v0.11.0 (Sepetmber 2020). Zenodo. 10.5281/ZENODO.592845

[B38] Weerdmeester J. van Rooij M. M. Engels R. C. Granic I. (2020). An integrative model for the effectiveness of biofeedback interventions for anxiety regulation: viewpoint. *J. Med. Internet Res*. 22:e14958. 10.2196/14958 32706654 PMC7413290

[B39] Wickens C. D. (2002). Multiple resources and performance prediction. *Theor. Issues Ergon. Sci.* 3 159–177. 10.1080/14639220210123806

[B40] Yu B. Funk M. Hu J. Wang Q. Feijs L. (2018). Biofeedback for everyday stress management: a systematic review. *Front. ICT* 5:23. 10.3389/fict.2018.00023

