# Supplementary material for: Breathing Biofeedback for Police Officers in a Stressful Virtual Environment: Challenges and Opportunities
Source: Front Psychol. 2021 Mar 12;12:586553. doi: 10.3389/fpsyg.2021.586553 (PMC7994769; doi:10.3389/fpsyg.2021.586553)
Supplement: Supplementary file 1 [file Data_Sheet_1.pdf]

Supplementary Material

1 Software and Hardware

We use a respiratory inductance plethysmography (RIP) belt[1] (Figure S1A) to acquire the analog breathing signal which is converted to a digital signal on a BITalino (r)evolution board[2] (Figure S1B). To obtain the biofeedback score, we then process the digital signal on a Raspberry Pi 4 Model B[3] (Figure S1C) with custom Python software using the open source EEGsynth library[4]. The biofeedback implementation can be found in the "biochill" module[5] of the EEGsynth library. See supplementary material B for details on the signal processing. The biofeedback score is then published to a Redis channel[6] (Figure S1D) that is monitored by a Unity VR engine[7] (Figure S1E) which renders the biofeedback representation in the virtual environment accordingly. Eventually, the virtual environment is presented using a HTC Vive headset[8] (Figure S1F). We would also like to point the reader to the Biocybernetic Loop Engine[9] as well as the Excite-O-Meter[10] as two additional software tools for integrating physiological measurements into a VR environment.

[1]https://www.biosignalsplux.com/products/sensors/respiration-inductive.html

[2]https://bitalino.com/en/plugged-kit-bt

[3]https://www.raspberrypi.org/products/raspberry-pi-4-model-b/

[4]https://scicrunch.org/resources/about/registry/SCR_018732

[5] https://github.com/eegsynth/eegsynth/master/module/biochill/biochill.py

[6]https://redis.io/

[7]https://unity.com/unity/features/vr

[8]https://www.vive.com/eu/product/#vive%20series

[9]https://doi.org/10.5220/0006429800450054

[10]https://sites.google.com/view/exciteometer/

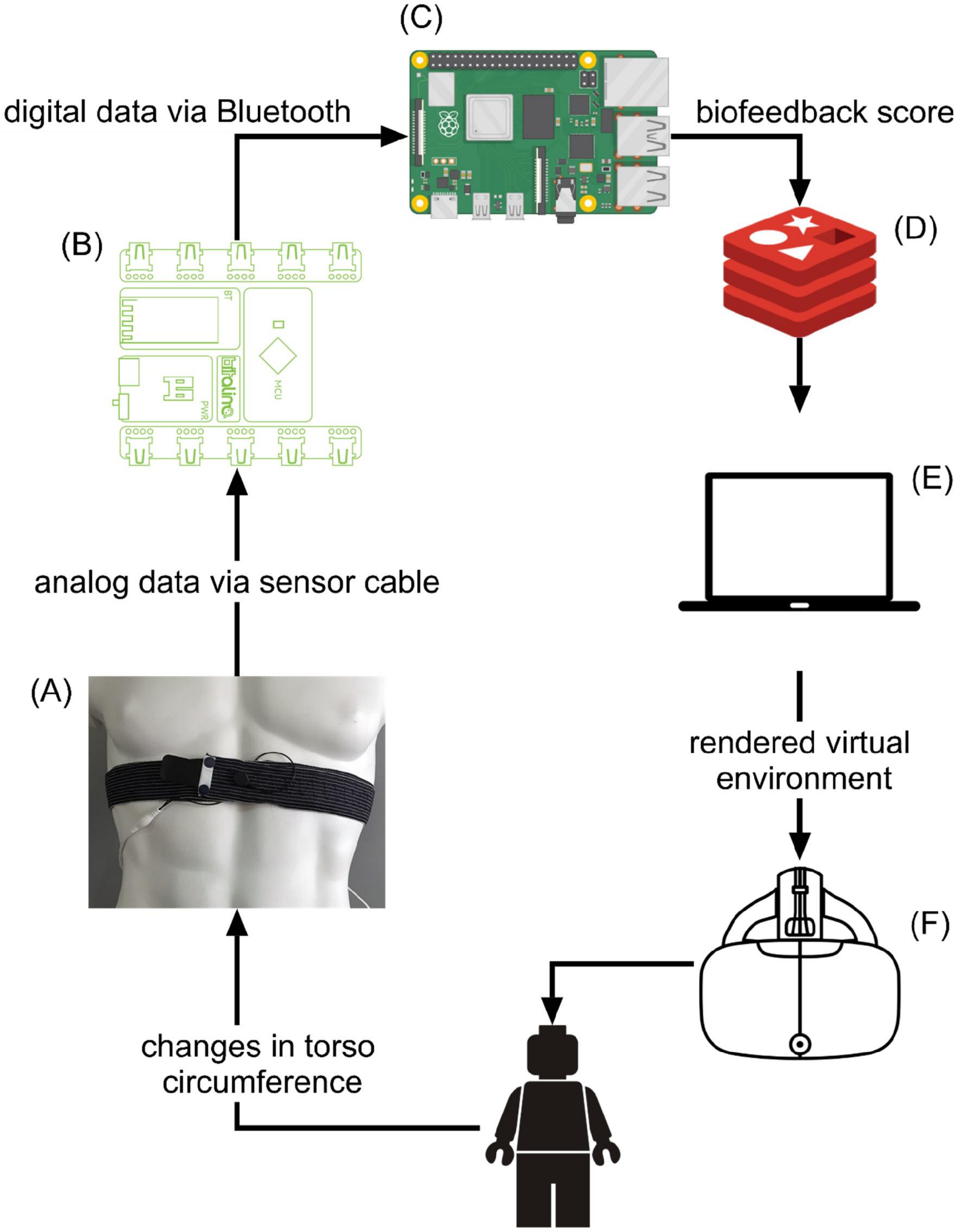

Figure S1. Components of the real-time biofeedback loop.

2 Signal Processing

In our real-time signal processing loop, we process 30 seconds of breathing data every two seconds (i.e., shifting window of 30 seconds with a hop size of 2 seconds and a resulting overlap of 28 seconds). We use spectral density estimation (SDE) to approximate the current breathing rate from each new breathing segment. SDE indicates how much of a certain frequency can be found in a signal. Breathing frequency is the number of breaths per second, similarly to breathing rate, the number of breaths per minute. A plot of spectral density (aka power spectrum) has power ("how much") on the y-axis and frequency ("how fast") on the x-axis.

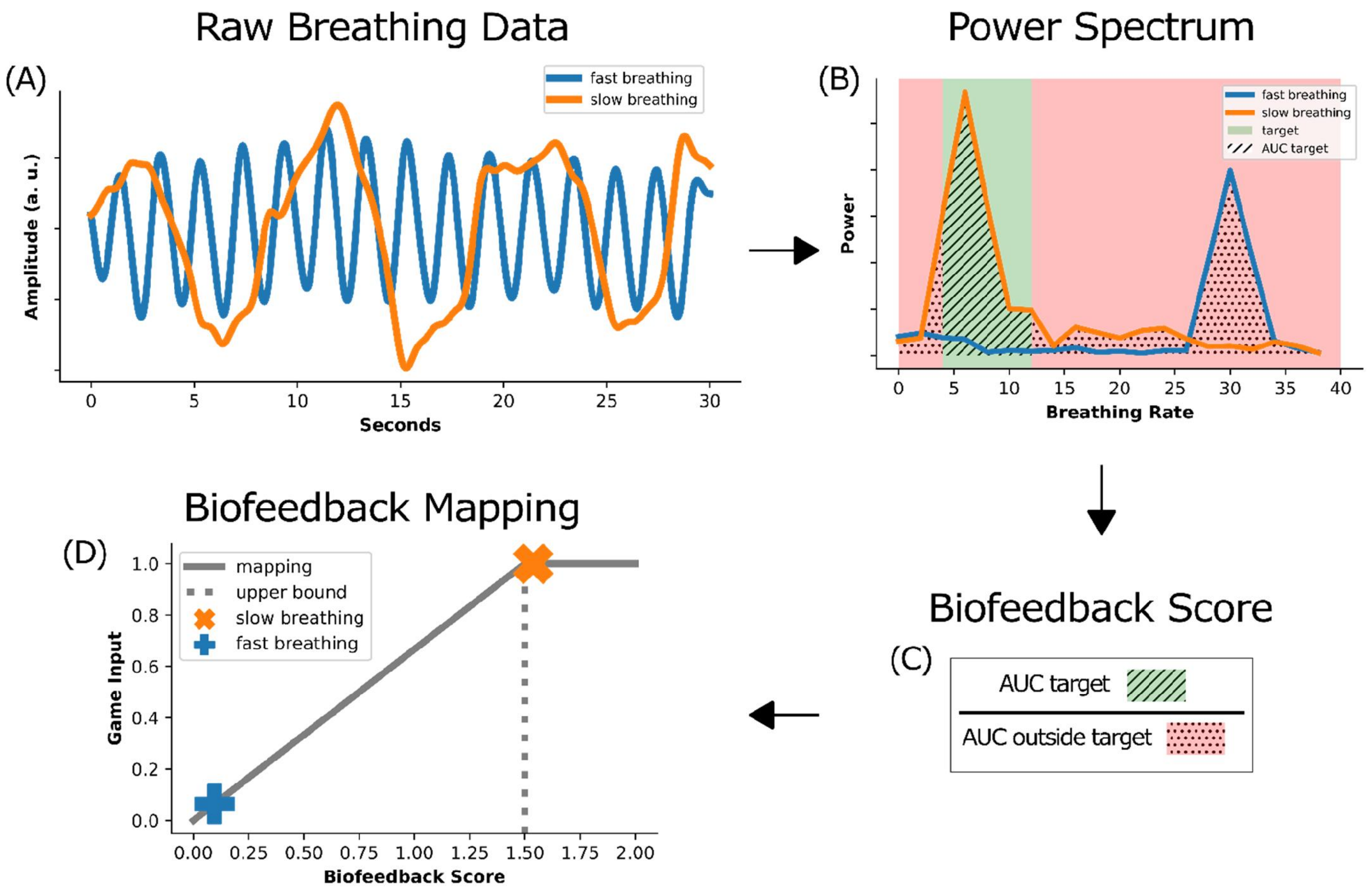

Figure S2. Sequence of biofeedback processing steps from raw data to game input. The blue and orange elements represent two breathing signals that are moved through the processing steps. The blue elements correspond to a signal with a fast breathing rate, the orange elements pertain to a signal with a slow breathing rate.

Figure S2B shows the power spectra of the two breathing signals in Figure S2A. The orange breathing signal contains a breathing rate of 6 breaths per minute whereas the blue breathing signal contains a breathing rate of 30 breaths per minute. We use the power spectrum to evaluate how much the current breathing rate matches the biofeedback target (range between 4 and 12 breaths per minute, green area in Figure S2B). We do so by integrating the area under the curve (AUC) of the power spectrum over the interval of the biofeedback target (striped area in Figure S2B). To obtain the biofeedback score, we divide this area by the remaining area under the power curve (dotted area in Figure S2B), which spans zero to 40 breaths per minute (red area in Figure S2B). In other words, the

biofeedback score is the ratio of a) spectral density over the biofeedback target range relative to b) the spectral density outside of the target range (Figure S2C). Since the biofeedback score is used to control elements of the virtual training environment (game input, as discussed in more detail in Challenge 3 of the main article), it is convenient to have the score be in a standardized range. We map the biofeedback score to a game input value ranging from 0 (worst) to 1 (best) (Figure S2D). There are two decisions regarding this mapping, which have important consequences for the trainee's experience of the biofeedback. First, we needed to define an upper bound on the ratio that represents the biofeedback score, and second, a transformation function has to be chosen. We chose an upper bound of 1.5 (dashed line in Figure S2D), which means that the trainee obtains the most rewarding biofeedback (game input = 1) when the spectral density within the biofeedback target range is at least 1.5 times larger than the spectral density outside of the target range. When the upper bound is set higher, it is more difficult for the trainee to obtain the most rewarding feedback. Ideally the upper bound would be adjusted to the current skill level of the trainee (e.g., raise the upper bound as the trainee gets more skilled at controlling their breathing, to keep the training challenging).

For the transformation function we chose a linear mapping of the biofeedback score to the game input (solid line in Figure S2D). This means that changes in the biofeedback score (up until the upper bound) will produce equivalent changes in the game input. In contrast, an exponential mapping produces more pronounced changes in the virtual environment at high values of the biofeedback score, and less pronounced changes at medium values. However, we want the biofeedback to be equally salient throughout its entire range.

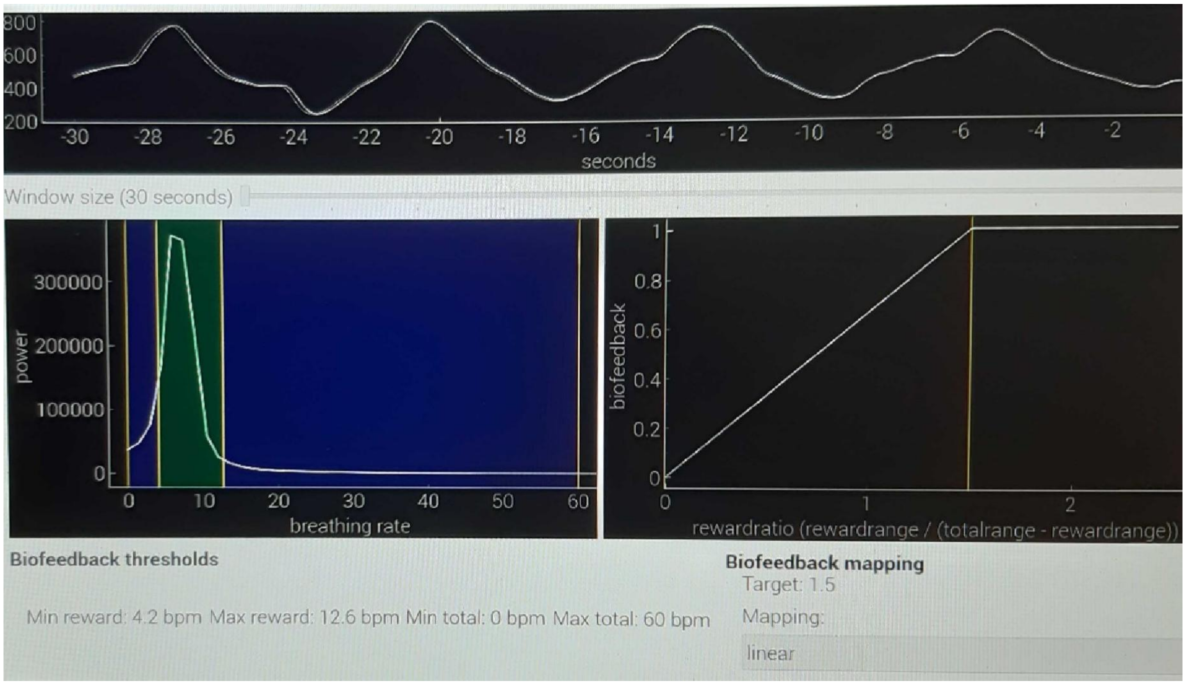

Figure S3. Biofeedback dashboard. The dashboard allows us to monitor the breathing signal and adjust a number of biofeedback processing parameters in real-time: 1. The length of the breathing signal that is processed every two seconds (upper panel). 2. The upper and lower breathing rate of the biofeedback target range and total range (lower left panel), 3. The kind of biofeedback mapping from biofeedback score to game input as well as the upper bound on the biofeedback score (lower right panel). The ability to adjust these parameters in real-time accelerates the process of homing in on a suitable biofeedback configuration during user testing.

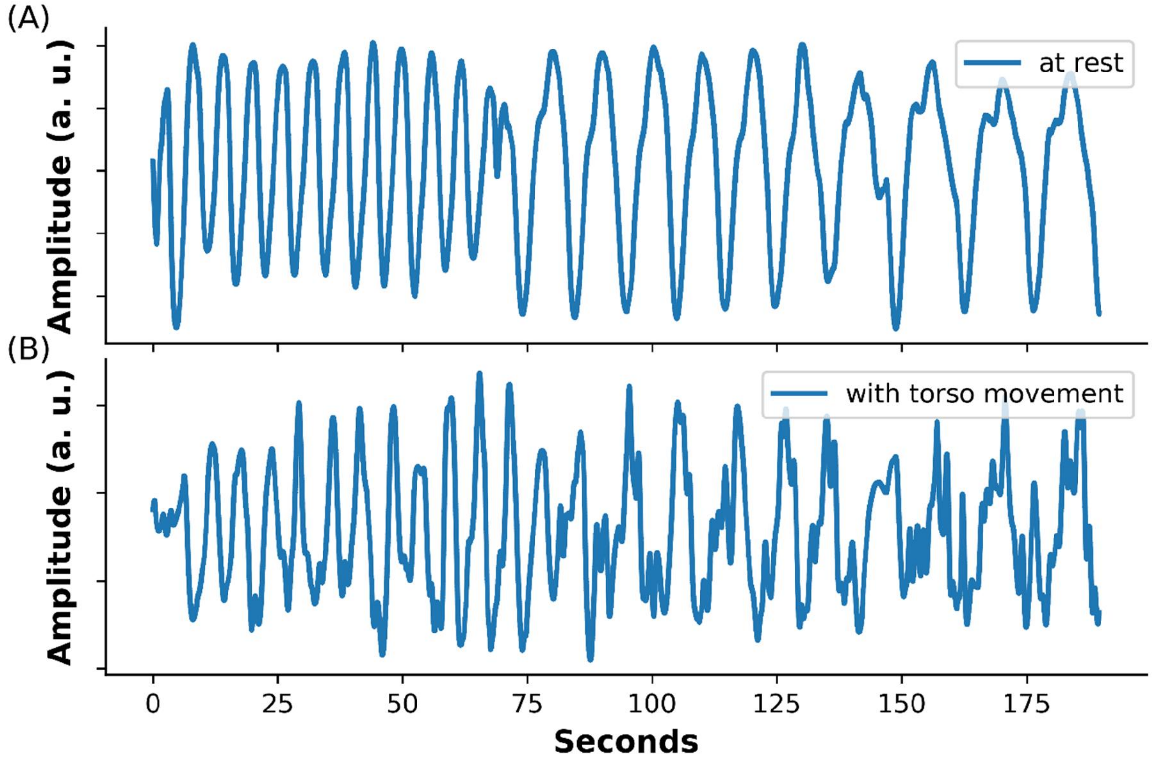

Figure S4. Artifacts in a breathing signal. A user is going through a paced breathing exercise, starting with a breathing rate of 10 breaths per minute (bpm), then shifting to 6 bpm, and finally breathing at 4 bpm. The exercise is conducted twice, while the user is standing in a relaxed position **(A)**, and while the user is standing and moving their torso **(B)**. Especially at lower breathing rates, the artifacts induced by the torso movement distort the signal. Note that the artifacts are in the frequency range of fast breathing.

3 Virtual Training Environment

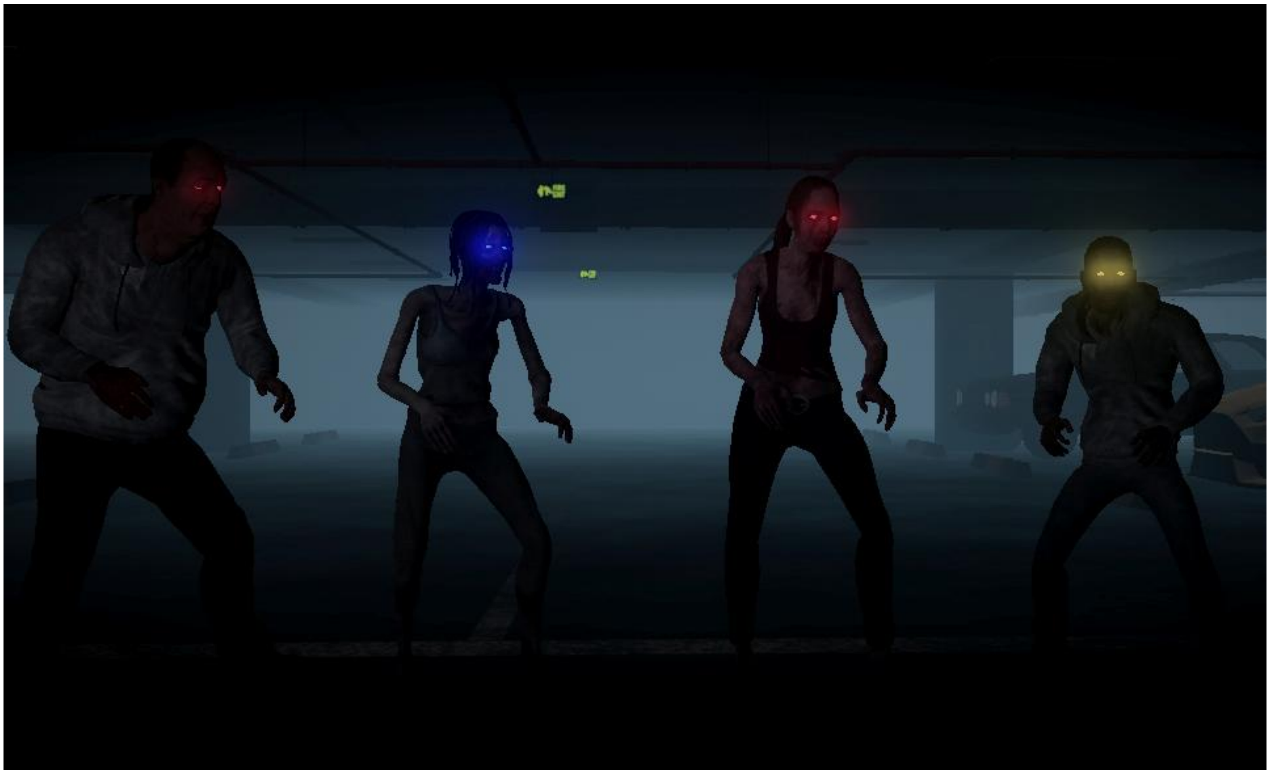

Figure S5. Avatar identifiers.

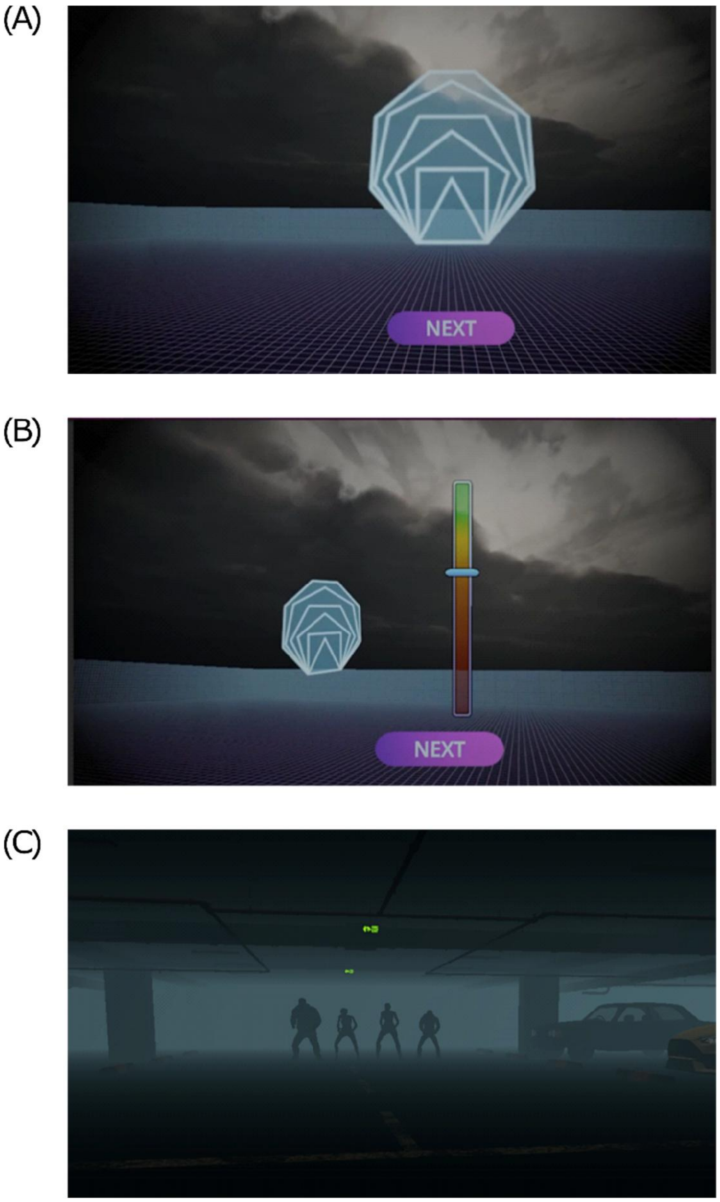

Figure S6. Breathing tutorial.
